# Causal relationship between Alzheimer’s disease and unstable angina: a bidirectional Mendelian randomization analysis

**DOI:** 10.3389/fpsyt.2024.1435394

**Published:** 2024-07-09

**Authors:** Yu-hang Chen, Cong-ying Ren, Cao Yu

**Affiliations:** ^1^ Department of Operations Management, Chongqing Mental Health Center, Chongqing, China; ^2^ Department of Hospital Infection Control, Chongqing Mental Health Center, Chongqing, China; ^3^ Department of Cardiothoracic Surgery, Chongqing University Jiangjin Hospital, Chongqing, China

**Keywords:** Alzheimer’s disease, cardiovascular diseases, coronary artery disease, unstable angina, Mendelian randomization, genome-wide association study, causal inference

## Abstract

**Background:**

Research from observational studies has demonstrated a link between Alzheimer’s disease (AD) and a higher risk of cardiovascular disease (CVD). Uncertainty surrounds the exact genetic cause of AD and coronary heart disease, particularly unstable angina (UA). Mendelian randomization (MR) analysis was used to examine the causal genetic link between AD and UA to evaluate the impact of AD on UA.

**Methods:**

The purpose of the bidirectional MR analysis was to investigate the link between exposure and illness causation. Genetic instrumental variables for AD were obtained from European populations using genome-wide association studies (GWAS). The primary causal conclusions were obtained using the inverse variance weighted approach (IVW), and other sensitivity analysis techniques were employed. Sensitivity analyses were carried out to evaluate heterogeneity and horizontal pleiotropy to guarantee accurate MR results.

**Results:**

An elevated risk of UA was linked to genetically predicted AD (IVW: OR=3.439, 95% CI: 1.565-7.555, P=0.002). A substantial genetic relationship between UA and the risk of AD was not supported by any evidence in the reverse study (IVW: OR=0.998, 95% CI: 0.995-1.001, P=0.190). Various MR techniques produced consistent results. Sensitivity analysis revealed no discernible heterogeneity or horizontal pleiotropy.

**Conclusions:**

One risk factor for UA that we found in our bidirectional Mendelian randomization trial was AD. This highlights the necessity of researching the underlying molecular mechanisms linked to AD and UA as well as the possibility of creating individualized treatment plans based on genetic data.

## Introduction

Alzheimer’s disease (AD) is a chronic neurodegenerative disease with an insidious onset and progressive progression. It is marked by a progressive loss of cognitive function and behavioral abilities (1). Around 10% of people over 65 and up to 50% of patients over 85 have AD, indicating an increasing prevalence of the disease with age (1). AD is the most prevalent type of dementia, making up about 60%–70% of all dementia cases. It affects over 55 million people globally, and the World Health Organization estimates that by 2050, there will be 152 million AD patients (2). With almost 9 million patients over 60 in China, AD has a major effect on families as well (3). Cognitive dysfunction caused by AD results in irreversible loss of self-care ability, and is characterized by a long duration of illness and many complications, thus requiring long-term family care, which undoubtedly brings great pressure on the family (4). AD also places a significant financial strain on society. The annual expense of treating this condition can reach $305 billion in the United States alone (5). Regretfully, the disease’s etiology is still unknown after decades of research conducted all around the world, which makes therapy challenging. In the treatment of AD, it is important not only to slow down the progression of dementia and reduce symptoms but also to focus on a range of complications to improve the quality of life of patients.

Cardiovascular disease (CVD) includes angina pectoris (AP), myocardial infarction (MI), atrial fibrillation (AF), heart failure (HF), and ischemic stroke (IS) and is currently the largest cause of death globally (6). As one of the representative diseases, unstable angina (UA) falls under the category of acute coronary syndromes. Despite the variety of available treatments, the pathological structure of the cardiac vasculature cannot be completely reversed. This well-known public health issue continues to be the leading cause of death worldwide, with the disease’s serious consequences being disability and death (7). Over the years, a large number of studies have been devoted to the evaluation of unstable angina and other variants of acute coronary syndromes to ensure that accurate diagnostic tools and the most effective treatments are realized (8). But it’s also crucial to recognize risk factors early on and take appropriate action.

Complex interactions exist between AD and CVD. AD (9) and vascular dementia (VD) (10) are linked to common cardiovascular illnesses such as coronary artery disease (CAD), heart failure, and stroke. Specifically, AP has been linked to a higher risk of AD. It is unclear how AP causes this association, but it can indirectly cause cerebral hypoperfusion by influencing cardiac output, which in turn helps form β-amyloid plaques and neurofibrillary tangles, two significant characteristics of AD (11, 12). Nonetheless, the causal link between CAD and AD risk is debatable because two meta-analyses produced radically different findings (13, 14). According to a different study, there is disagreement regarding the results of research on the connection between AD and UA because of the presence of confounders, which can create spurious relationships. It is also unclear whether shared risk factors are the cause of the association (15).

Thus, more extensive research is required to support the idea that AD and UA risk are causally related. However observational studies have inherent flaws that may lead to biased results; randomized controlled trials are difficult to conduct because both diseases share risk factors like obesity, smoking, diabetes, and metabolic syndrome (16) and have a chronic course that necessitates long-term follow-up. Investigating the causative link between AD and UA is essential to better patient management and, ultimately, the patient’s clinical outcome in addition to assisting in the customization of the right treatment plan for the patient.

Genetic variation is used as an instrumental variable (IV) in Mendelian randomization (MR), a unique analytical technique for epidemiological investigations, to infer causal links between exposure factors and outcomes (17, 18). To prevent confounding variables and reverse causation, this method mimics the randomization procedure used in randomized controlled trials (RCTs) (19, 20). Many single nucleotide polymorphisms (SNPs) linked to AD and UA have been found through large-scale GWAS, and they largely follow the natural order of causality and offer a chance to investigate any potential causal relationships between the two. Furthermore, MR is a natural analog of RCTs due to the random assignment of genetic variants at meiosis, which lowers the possibility of bias in comparison to observational research (21). Due to the lack of clarity around the relationship between AD and UA, we used a two-sample MR analysis in this work and used SNPs as instrumental variables to investigate the bidirectional causality between AD and UA and offer fresh insights into the prevention and treatment of disease.

## Materials and methods

### Study design

**Figure 1:**
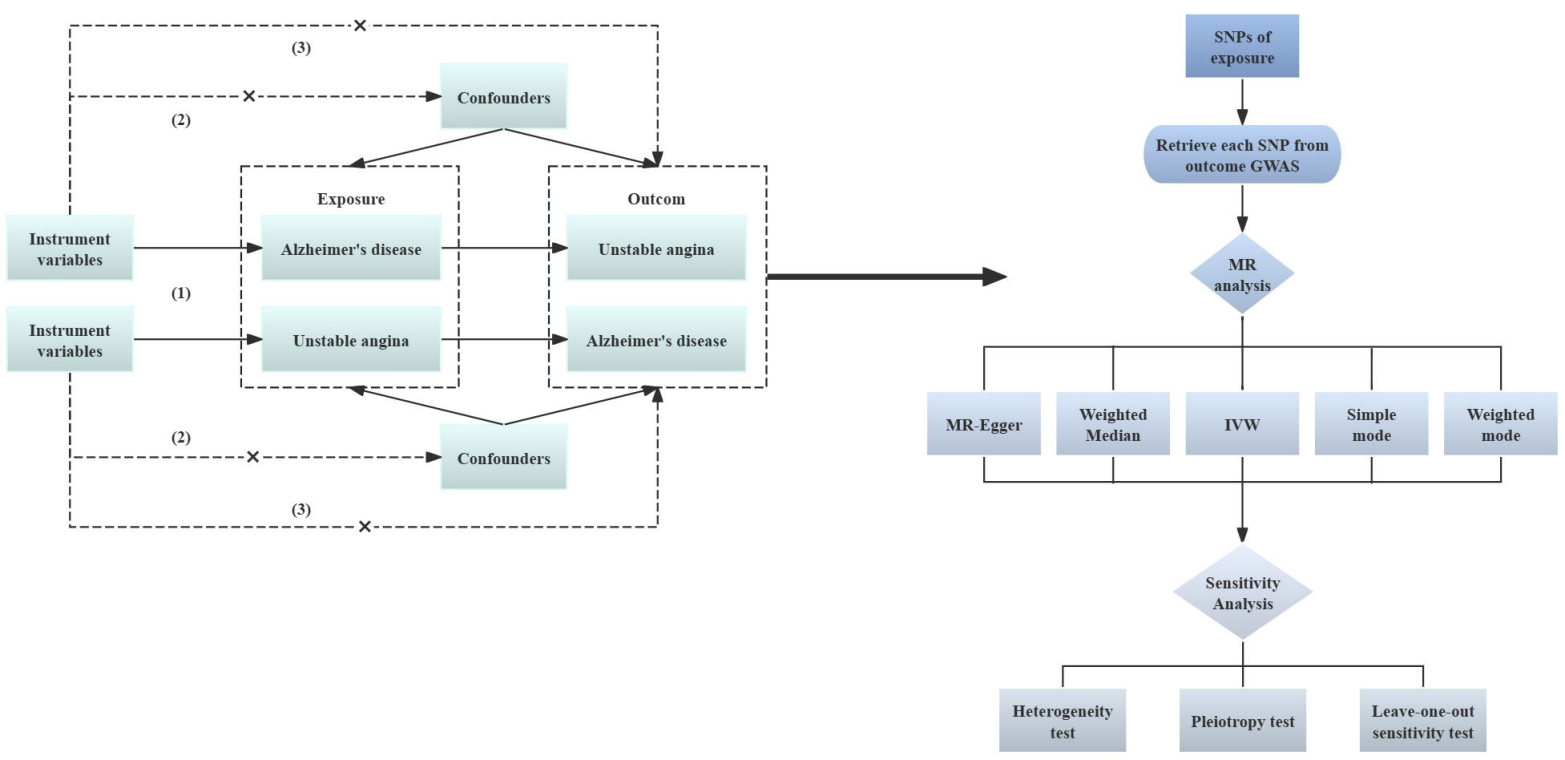
Analysis flow of two-sample MR. Solid lines indicate the presence of an association, dashed lines indicate the absence of an association; GWAS, Genome-wide Association Study; MR, Mendelian randomization; SNP, single nucleotide polymorphism; IVM, inverse variance weighted.

To investigate the causal association between Alzheimer’s disease and unstable angina, this study used a bidirectional two-sample Mendelian randomization technique. A pooled dataset from genome-wide association research was used for MR analysis, with AD serving as the “exposure” and UA as the “outcome.” An inverse variance weighting method was also used to determine the causal relationship between exposure and result. Three primary hypotheses are required for MR research (22, 23): (1) Correlation hypothesis (Hypothesis 1): Bidirectional two-sample Mendelian randomization analysis should be used to ascertain the link between the exposure and the result. Selected SNPs should exhibit a significant correlation with the exposure (Alzheimer’s disease). Hypothesis 2: Independence Hypothesis: The SNPs should be unaffected by potential confounders that may exist between the exposure and the result, in this study, unstable angina. (3) Exclusivity hypothesis (hypothesis 3): SNPs that are substantially linked to exposure are only causally linked through exposure; they are not directly related to outcome. **Figure 1** provides a summary of the study design.

### Data sources

The two samples used in the MR study have to come from the same population in order to reduce bias (24). The following website was used to gather pertinent genome-wide association study (GWAS) datasets: https://gwas.mrcieu.ac.uk. We proposed to select the largest sample size Alzheimer’s disease dataset from this website. However, each of the three recent datasets on Alzheimer’s disease suffered from the following problems: (1) the number of people in the case group was much lower than that of the control group, which made the analysis incomplete (ieu-b-5067); (2) the sum of the number of people in the case group and the control group was not equal to the sample size (ebi-a-GCST90027158); (3) there was no clear picture of the subgroup data (ebi-a-GCST90012877). Therefore, we finally chose the 2018 dataset (ID: ukb-b-14699), which has a large sample size and clearly describes the number of people in the case and control groups, as well as the dataset from the MRC-IEU, which provides accuracy and authenticity. We used the same principles to select the most recent unstable angina dataset with the largest sample size.

The GWAS dataset of exposure (ID: ukb-b-14699) containing 423,738 participants (36,548 Alzheimer’s disease sufferers and 387,190 control subjects) and roughly 9,851,867 SNPs sites were exclusively composed of European individuals. This data was obtained from the MRC-IEU and is the outcome of an analysis of the UK Biobank dataset by Ben Elsworth et al. With 24,179,929 SNPs, the GWAS dataset of outcome GWAS dataset (ID: ebi-a-GCST90018932), which was also from Europe, included 456,468 persons in total (of which 9,481 patients had unstable angina and 446,987 were control subjects). Comprehensive details are given in **Table 1**.

**Table 1:** Data description of Alzheimer’s diseases and Unstable angina.

Traits	Data source	PMID	Year	Sample size (cases/controls)	GWAS ID
Alzheimer’s disease/dementia	Ben Elsworth		2018	36548/387190	ukb-b-14699
Unstable angina	Sakaue S	34594039	2021	9481/446987	ebi-a-GCST90018932

### Screening of instrumental variables

First, more SNPs closely linked to Alzheimer’s disease were screened to test the correlation hypothesis. Typically, SNPs with P< 5×10-8 are considered genome-wide significant, but not enough SNPs were screened at this threshold. Therefore, this study used a more lenient threshold, setting P< 5×10-6 to screen significant SNPs (25, 26). The R software package “TwoSampleMR” was used to carry out the clump stage (27, 28). To remove the chain imbalance relationship and guarantee the independence of the screened SNPs, the parameters r2 = 0.001 and region width kb=10000 were specified (29). Second, the screened SNPs were searched in LDtrait (https://ldlink.nci.nih.gov/?tab=home), an open-access web-based tool for identifying germline variants linked to multiple traits, to confirm if the chosen instrumental variables met the independence and exclusivity assumptions (30). Five SNPs linked to confounders and ending variables (rs7223593, rs76856627, rs117310449, rs8106813, rs62119261) were carefully removed. Then, instrumental variable strength was assessed by calculating the F-value of individual SNP to exclude possible weak instrumental variable bias between instrumental variables and exposure with the following formula (31):


$$R^{2}=\frac{2\times MAF\times \left(1-MAF\right)\times \beta^{2}}{SE^{2}\times N}$$

$$F-statistic=\frac{R^{2}\times \left(N-k-1\right)}{k^{2}\left(1-R^{2}\right)}$$

A correlation between instrumental variables and exposure that is sufficiently high and minimizes the likelihood of weak instrumental variable bias is indicated by an F-statistic value larger than 10 (32). Eventually, the exposure and outcome datasets were merged, the palindromic SNPs in the merged dataset were eliminated, and the remaining SNPs were the instrumental variables for the MR analyses that followed.

### Statistical analysis

The Mendelian randomization analysis was carried out with the R software “TwoSampleMR” package. The key outcome was inverse variance weighting (IVW) analysis, which has significant efficacy in detecting causal relationships since it is predicated on the idea that the tool only influences outcomes as a result of exposure and not through other pathways (33). Even though this analysis eliminated as many SNPs known to be related to confounders as possible, there are still a lot of unidentified confounders that could skew the results. Consequently, in addition to the IVW analysis results, other analytical techniques such as the MR-Egger method, weighted median, weighted mode, and simple mode were also employed (34). IVW studies are most reliable when there is no horizontal pleiotropy in the instrumental variables. IVW combines the MR effect estimates of individual SNP to determine the possible causal effect of the overall weighted estimates (35, 36). According to research, the weighted median approach produces reliable estimates of causal effects even when up to 50% of the data is derived from genetic variation in the null instrumental variable (37). MR-Egger regression confirms whether there is horizontal pleiotropy in the instrumental variables, and when there is horizontal pleiotropy in the instrumental variables, MR-Egger regression still yields unbiased estimates of causality (38). Although the test efficacy of the simple mode is lower than that of the IVW method, it is robust against pleiotropy (39). Weighted mode is sensitive to the selection of bandwidth for model estimation (40).

IVW may be subject to bias or multiple effects due to null IVs, so the validity and robustness of the outcome were tested through a series of sensitivity analyses. Sensitivity analysis used Cochran’s Q test (41) to measure the heterogeneity of individual genetic variance estimates; a P>0.05 result for Cochran’s Q test meant that there was no heterogeneity among SNPs. By using the MR-Egger-intercept to test for potential horizontal pleiotropy (38), it was determined that there was no horizontal pleiotropy in the study if P>0.05. Sensitivity analysis using leave-one-out was also employed to determine the degree to which individual SNP affected causation following their elimination one by one from the final inclusion of SNPs. In addition, the statistical power of the MR analysis was calculated through an online tool (https://shiny.cnsgenomics.com/mRnd/).

## Results

### Instrumental variables

35 SNPs were screened and ultimately identified as instrumental variables using Alzheimer’s disease as the exposure factor and unstable angina as the outcome variable. The F-value calculation revealed that the maximum F-value was 1,737.714 the minimum F-value was 20.860, and the F-value of each SNP was greater than 10, indicating that the phenotype of AD and UA-related phenotypes was less likely to be impacted by the bias of weak instrumental variables.

### MR estimates and sensitivity analyses

**Figure 2:**
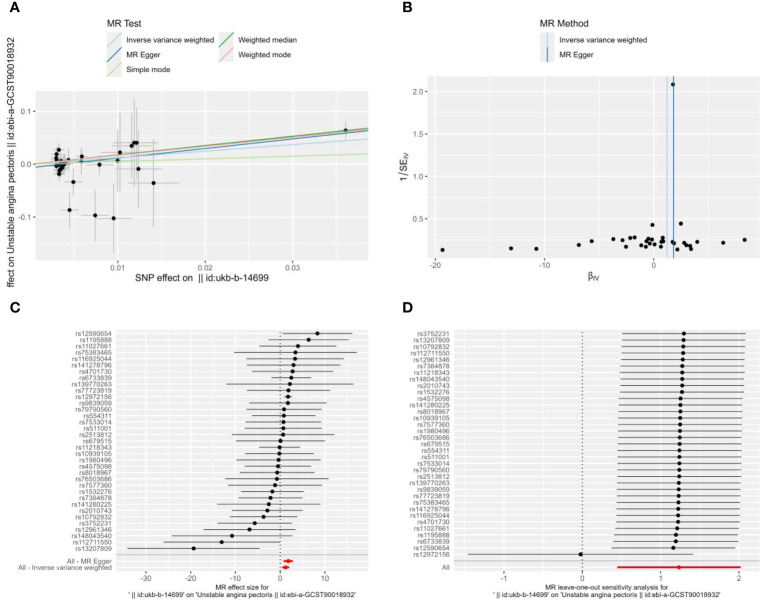
Scatter plot **(A)**, funnel plot **(B)**, forest plot **(C)**, and leave-one-out analysis **(D)** of the effect of Alzheimer’s disease on Unstable angina(UA). The lines in **(A)** illustrate the estimated effect sizes by MR methods. **(B)** demonstrates that the funnel plot is symmetric, which indicates that the MR estimates are reliable. **(C)** shows the MR estimate of each SNP effect on UA. **(D)** depicts the changes in MR estimates after excluding individual SNP.

The MR-Egger regression’s intercept in the current investigation was nearly 0 (intercept=-0.007, P=0.102), suggesting that the instrumental variables lacked horizontal pleiotropy (**Figure 2A**). As seen in **Figures 2A, C** with **Table 2**, MR analysis with IVW as the primary analysis method revealed a causal link between Alzheimer’s disease and an elevated risk of unstable angina (OR=3.439, 95% CI: 1.565-7.555, P=0.002).

**Table 2:** The Mendelian randomization of Alzheimer’s disease and Unstable angina.

Exposure	Outcome	SNPs	Method	OR	95%CI	*P*
Alzheimer’s disease	Unstable angina	35	MR-Egger	6.128	(2.173-17.284)	0.002
			weighted median	5.845	(2.323-14.7703)	<0.001
			IVW	3.439	(1.565-7.555)	0.002
			simple mode	1.656	(0.080-34.404)	0.746
			weighted mode	5.472	(2.176-13.764)	<0.001
Unstable angina	Alzheimer’s disease	17	MR-Egger	0.999	(0.991-1.008)	0.901
			weighted median	0.999	(0.994-1.003)	0.585
			IVW	0.998	(0.995-1.001)	0.190
			simple mode	1.001	(0.993-1.009)	0.767
			weighted mode	0.999	(0.994-1.005)	0.858

SNPs, single nucleotide polymorphisms; OR, odds ratio; CI, confidence interval; IVW, inverse-variance weighted.

Heterogeneity between instrumental variables was detected using IVW and MR-Egger regression. The results of MR-Egger regression showed that Cochran’s Q=30.221, Q_df=33, P=0.606; the results of IVW showed that Cochran’s Q=33.036, Q_df=34, P=0.515 (**Figure 2B**); this indicates that there was no heterogeneity among the instrumental variables.

To ascertain whether the causal associations were caused by a single instrumental variable, sensitivity analyses were carried out using the leave-one-out method, in which SNP was eliminated one at a time. The causal effects of the remaining SNPs were then compared with the findings of the MR analyses of all SNPs. The robustness of the SNPs analysis was demonstrated by the results of the sensitivity analyses (**Figure 2D**).

Subsequently, we further calculated the statistical power of the MR analysis. The outcome sample size for this study was 456,468, the proportion of cases in the study was K=0.021, R2 = 0.006, OR=3.439, and the statistical power calculated by the online tool was 1.00. Therefore, it is unlikely that this study will have a false- positive result, and based on the results of the IVW method, it can be concluded that there is a causal association between Alzheimer’s disease and unstable angina.

### Reverse direction analysis

**Figure 3:**
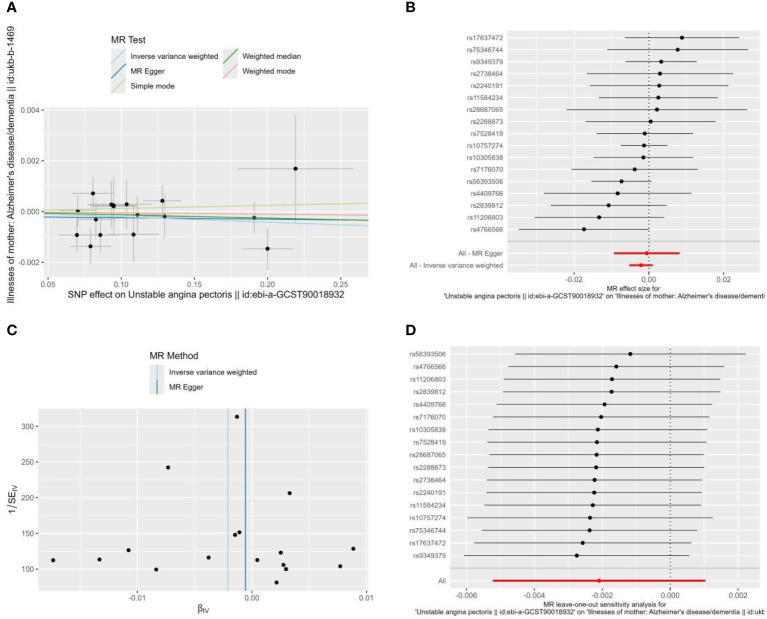
Scatter plot **(A)**, Forest plot **(B)**, funnel plot **(C)**, and leave-one-out analysis **(D)** of the effect of Unstable angina on Alzheimer’s disease (AD). The lines in **(A)** illustrate the estimated effect sizes by MR methods. **(D)** shows the changes in MR estimates after excluding each individual SNP.

To determine whether reverse causation exists, we also performed a reverse MR study using unstable angina as an exposure factor and Alzheimer’s disease as an outcome variable. According to **Figure 3A**, the horizontal multivariate validity test revealed that the instrumental variables had no horizontal multivariate validity (intercept=-0.0001, P=0.722). When using IVW, OR=0.998, 95% CI: 0.995-1.001, P=0.190, the MR analyses’ findings demonstrated that there was no meaningful causal relationship between UA and the risk of AD (**Table 2**, **Figure 3B**).

There was no heterogeneity among the instrumental variables in the heterogeneity test (**Figure 3C**), as indicated by the MR-Egger regression findings that produced Cochran’s Q=13.038, Q_df=15, P=0.599, and the IVW results that produced Cochran’s Q=13.170, Q_df=16, P=0.660. For MR analysis, sensitivity analyses produced trustworthy results (**Figure 3D**).

## Discussion

### Principal findings

In this study, we applied MR analysis for the first time, extracting GWAS data on unstable angina and Alzheimer’s disease in a European population. We discovered that there was no causal relationship between unstable angina and increased risk of Alzheimer’s disease (OR=0.998, 95% CI: 0.995-1.001, P=0.190), but there was a causal relationship between Alzheimer’s disease and increased risk of unstable angina (IVW: OR=3.439, 95% CI: 1.565-7.555, P=0.002).

### Possible mechanisms

Over the past few decades, there has been a significant expansion and deepening of our understanding of the interactions between the heart and the brain. The heart and brain are not isolated systems but fundamentally interconnected by forming neurovascular and humoral pathways, called the heart-brain axis. Alzheimer’s disease and cardiovascular disease may occur as a result of abnormalities in the heart-brain axis (42). Smoking, diabetes, and hypertension have all been identified as risk factors for dementia and cardiovascular disease in earlier research (43–45). Coronary heart disease (CHD) is a significant cardiovascular illness that has been proven to be highly related to dementia in recent years [46]. Adults with CHD experience rapid cognitive deterioration after an episode of the disease. Meta-analyses have shown evidence that suggests a higher risk of dementia is present in people with coronary heart disease (46). This view is further supported by a large longitudinal population-based cohort study that uses data from the UK Biobank and, more importantly, shows that the younger the age of onset of coronary heart disease, the higher the risk of dementia; that is, the strength of the association between dementia events and coronary heart disease increases progressively as age of onset decreases (47).

Alzheimer’s disease is primarily caused by inflammation (48). In the brain, β-amyloid deposition, neurofibrillary tangles, and neurotoxic peptide aggregation trigger inflammatory pathways and lead to the build-up of inflammatory mediators such as cytokines, carotenoids, and others that cause neuroinflammation (49–51). The amyloid hypothesis of Alzheimer’s disease is characterized by β-amyloid (Aβ), which is thought to be a result of impaired perivascular deposition drainage in the walls of tiny arteries. Aβ1–40 is the primary peptide implicated in pathogenesis among them; due to its degree of vascular preference, this molecule can be found in the peripheral vascular system as well as the cerebrovascular system, where it may mediate arterial illness by exerting pro-inflammatory effects (52).

Angiographically verified coronary artery disease (CAD) was independently correlated with circulating Aβ1-40 levels in a 2-group independent cohort study with 514 versus 396 participants (53). There is evidence to show that Aβ is concentration-dependent on the severity of acute coronary syndrome (ACS) (54) and that it may play a direct role in plaque rupture and thrombosis, which in turn generate the usual clinical signs of ACS (52).

Past research indicates that the APOE4 gene plays a role in the pathophysiology of AD. It may work by competitively binding to low-density lipoprotein receptor-associated protein 1, which inhibits the clearance of Aβ and raises the risk of AD by accumulating Aβ in the brain. It also partially regulates blood Aβ hemodynamics (55). The protein that this gene codes for is also involved in the metabolism of lipoproteins, such as triglycerides and cholesterol, and this process aids in the development of atherosclerosis, a condition that is known to be a major cause of coronary artery disease (56).

According to the results of the present study, rs6733839 may be a bridge that closely links Alzheimer’s disease to unstable angina. rs6733839 is located near the bridging integrator 1 (BIN1) gene, which in turn affects the accumulation of the two major pathological hallmarks of AD, namely β-amyloid and Tau (57). BIN1 has also been found to be a regulator of transverse tubule function and calcium signaling in cardiomyocytes, and is associated with abnormal cardiac contraction, increasing the likelihood of malignant arrhythmias before heart failure (58); plasma levels of cardiac bridging integrator 1 (cBIN1) also indicative of the effects of coronary microvascular dysfunction on cardiomyocytes (59). Thus, this may be a potential mechanism for the relationship between the two diseases.

Without a doubt, there is some degree of relationship between AD and UA because they have similar molecular mechanisms and linking pathways, in addition to sharing risk factors. By comprehending the causes and mechanisms of action of these two illnesses, novel therapeutic approaches for clinical management may be developed. To better understand this relationship and develop a more effective treatment plan for patients with AD and related dementias as well as UA, more research is required. This research should include the use of cutting-edge neuroimaging, cardiac and neurological biomarkers, proteomics, and metabolomics technologies.

### Strengths and limitations

This study used two-sample MR to investigate the causal relationship between AD and UA risk factors. Its main contributions are as follows: first, it provides a broad reference for future research into the etiology and mechanism of the disease, as well as for the development of interventions, diagnostics, and therapeutic measures. Secondly, by building MR models to investigate the etiology of the disease, this study circumvents the effects of reverse causation and confounding variables, which are insurmountable in conventional observational investigations.

This study has certain limitations as well. First, since all GWAS data came from European populations, it is still unclear if the results apply to other populations. Secondly, subgroup analysis was not possible to determine causal associations following precise categorization since the available GWAS data did not include comprehensive clinical information. Additionally, the nonlinear relationship between exposure and outcome could not be assessed because the current study used pooled GWAS data.

## Conclusion

We offer evidence for a potential causal link between AD and UA through Mendelian randomization analysis. The risk of UA is increased more when AD is present. The genetic similarities between AD and UA may offer important new information for the creation of preventative and therapeutic measures. To comprehend the molecular mechanisms underlying this association and investigate customized treatments based on genetic data, more research is required.

## Data availability statement

The datasets presented in this study can be found in online repositories. The names of the repository/repositories and accession number(s) can be found in the article/Supplementary Material.

## Author contributions

YC: Data curation, Methodology, Software, Writing – original draft, Investigation. CR: Investigation, Writing – review & editing. CY: Validation, Writing – review & editing.
